# 

**Published:** 1834-07-01

**Authors:** 


### MEDICO-CHIRURGICAL

### REVIEW,

AND

### JOURNAL

OF

### PRACTICAL MEDICINE.

*(NEW SERIES.)*

VOLUME TWENTY-ONE,

[1st of APRIL to 30th of SEPTEMBER,]

1834.

VOL. I. of DECENNIAL SERIES.

EDITED

BY JAMES JOHNSON, M. D.

PHYSICIAN EXTRAORDINARY TO THE KING,

AND

HENRY JAMES JOHNSON, ESQ.

LATE HOUSE SURGEON TO ST. GEORGE'S AND THE LOCK HOSPITALS.

57461
LIBRARY.
Washington, D.C.
not to be indexed

LONDON:

PUBLISHED BY S. HIGHLEY, 32, FLEET STREET,

*Re-printed in New York, by Mr. Wood.*

1834.

W1
ME 75 T

PRINTED BY G. HAYDEN,

Little College Street, Westminster.

### BIBLIOGRAPHICAL RECORD;

OR,

*Works received for Review since last Quarter.*

1. Medica Sacra; or, Short Expositions of the more important Diseases mentioned in the Sacred Writings. By THOMAS SHAPTER, M.D. Physician to the Exeter Dispensary, &c. Small octavo, pp. 191. Longman and Co. 1834. Price 7s.

---

2. Observations on the Preservation of Sight, and on the Use, Abuse, and Choice of Spectacles, Reading-glasses, &c. By JOHN HARRISON CURTIS, Esq. Oculist and Aurist. Octavo, pp. 48. Highley, 1834.

☞ *This little work should be in the hands of all young people, and would thus save annually many valuable eyes.*

---

3. A Letter to Henry Warburton, Esq. M.P. on the Grievances affecting the Medical Profession. By a Junior Practitioner. Octavo, pp. 48. Churchill, March, 1834.

☞ *A very good digest of the ills which press on medical society.*

---

4. Six Introductory Lectures on the Institutes and Practice of Medicine. By BENJAMIN RUSH, M.D. Philadelphia, 1801.

---

5. The Philosophy of the Human Voice, embracing its Physiological History; together with a System of Principles by which Criticism on the Art of Elocution may be rendered intelligible, and Instruction definite and comprehensive, &c. &c. By JAMES RUSH, M. D. Second Edition, enlarged. Philadelphia, 1833.

☞ *This is a very talented and elaborate treatise on the subject which it embraces.*

---

6. The Physiology, Pathology, and Treatment of Asphyxia; including Suspended Animation in New-born Children, Drowning, Hanging, Wounds of the Chest, &c. &c. By JAMES PHILLIPS KAY, M.D. Octavo, pp. 344. Longman and Co. March 1834.

---

7. Suggestions respecting the intended Plan of Medical Reform, &c. By JOS. HENRY GREEN, F.R.S., F.G S. &c. Octavo, pp. 49. Highley, 32, Fleet Street. March, 1834.

8. Outlines of the Anatomy and Physiology of the Teeth, &c., their Diseases, and Treatment, &c. By DAVID WEMYS JOBSON, M.R.C.S. &c. Octavo, pp. 268, with plates. Edinb. and London, 1834.

---

9. Hippopathology; a Systematic Treatise on the Disorders and Lamenesses of the Horse, with their modern and most approved Methods of Cure, &c. &c. By WILLIAM PERCIVAL, M.R.C.S. Veterinary Surgeon in the First Life Guards. Vol. 1. Octavo, pp. 331. Longman and Co. 1834.

☞ *Reviewed.*

---

10. The Principles and Practice of Obstetric Medicine, &c. &c. By Dr. DAVIS. Parts 29, 30, 31, 32. 1834. 2s. each. Malignant Diseases of the Uterus.

---

11. Encyclopédie des Sciences Medicales, Premiere Division, Anatomie et Physiologie. Premiere, deuxieme et troisieme livraison. Paris, 1834.

☞ *Each livraison contains about* 130 *or* 140 *pages of close type in double columns. It is to be to comprized in* 100 *livraisons, or* 25 *volumes, in monthly numbers, one franc and a half each. The work is remarkably cheap, and is on a novel plan. It is edited with great ability, if we can judge by the first three livraisons.*

---

12. A Series of Anatomical Plates, in Lithography, &c. &c. By JONES QUAIN, M.D. Fasciculi XI. XII. XIII. & XIV. Division, Muscles. April, May, June, 1834, price two shillings each, folio.

---

13. Pathological and Surgical Observations on the Diseases of the Joints. By B. C. BRODIE, V.P.R.S. Serjeant-Surgeon to the King, and Surgeon to St. George's Hospital. Third Edition, with Alterations and Additions. Octavo, pp. 344. London, 1834.

---

14. The Signs, Disorders, and Management of Pregnancy:—the treatment to be adopted during and after Confinement; and the Management and Disorders of Children. Written expressly for the use of Females. By Douglas Fox, M.R.C.S. and one of the Surgeons to the Derbyshire General Infirmary. Octavo pp. 206. Derby, April, 1834.

☞ *This work is plainly and delicately written. It is better calculated for the object which it has in view, than any work of the kind with which we are acquainted.*

---

15. Essai sur la Leucorrhée et les Causes diverses qui la produisent. Par A. M. Reofrey, M.D. Octavo, pp. 161. Prix 3s. 6d. A Londres, chez l'Auteur, Newman Street, J. B. Bailliere, April, 1834.

☞ *This is a sensible little work in which the various causes of leucorrhœa are traced to their sources, with numerous cases in illustration.*

---

16. Hortus Medicus, or Figures and Descriptions of the more important Plants used in Medicine, or possessed of Poisonous Qualities; with their Medical Properties, Chemical Analyses, &c. &c. By George Graves, M.D. Fellow of the Royal College of Physicians of Edinburgh, &c. &c. &c. Quarto, pp. 272, with 44 plates and numerous figures. Edinb. & London, 1834.

---

17. Sur la Pathogénie et quelques Affections de l'Axe Cerebro-spinal, &c. Choix d'Observations prises dans l'Hôpital de Bourdeaux. Par Rey, (M.L.) M.D. Chirurgien chef interne de l'Hopital Saint-Andre de Bourdeaux. Avec une planche. Quarto, pp. 111. Bailliere, Regent Street, 1834. Price three shillings.

---

18. The Anatomy of the Human Eye. By John Dalrymple, Assistant-Surgeon to the London Ophthalmic Infirmary. 8vo. pp. 294, with plates. Longman and Co. April 1834.

---

19. The Principles of Physiology applied to the Preservation of health, and to the Improvement of Physical and Mental Education. By Andrew Combe, M.D. Fellow of the Royal College of Physicians, Edinb. Octavo, pp. 320. Edinburgh and London. May, 1834.

---

20. The Principles and Practice of Obstetricy, as at present taught. By James Blundell, M.D. Professor of Obstetricy at Guy's Hospital, &c. &c. &c. Edited by Thomas Castle, M.D. F.L.S. Octavo, pp. 838. Cox and Son. May, 1834.

☞ *This is one of the most valuable works on midwifery that we have yet seen. The talents and experience of the author stamp it with the seal of sterling merit. We shall notice it analytically in our next.*

---

21. The Transactions of the Provincial Medical and Surgical Association. Instituted 1832. Volume the Second. 1834. Pp. 346, with plates.

☞ *This volume came to hand too late to be completely noticed in the present number of the Journal; but we shall not fail to make its valuable contents fully known to our readers, in our next.*

---

22. A Treatise on Epidemic Cholera; illustrating a new Theory of the Disease, on which the Principles of a systematic Mode of Treatment are established. By Jacob Vale Asbury, M.R.C.S. &c. &c. Octavo, pp. 55.

---

23. An Account of Jane C. Rider, the Springfield Somnambulist; the substance of which was delivered as a Lecture before the Springfield Lyceum, Jan. 22d, 1834. By L. W. Belden, M.D. Duodecimo, pp. 134. April 1834.

---

24. On the Necessity of Popular Education, as a National Object; with Hints on the Treatment of Criminals, and Observations on Homicidal Insanity. By James Simpson, Advocate. Octavo, pp. 402. Edinburgh, 1834.

☞ *We shall notice some parts of this valuable little work in our next.*

---

25. An Introductory Lecture to the Practice of Physic, at the Opening of the Eccles Street School of Medicine and Surgery. By W. Stoker, M.D.

---

26. A Letter addressed to the Medical Practitioners of Great Britain on the subject of Homœopathy. By the Rev. Thomas Everest, Rector of Wiekar, Gloucestershire. Octavo, stitched, pp. 40. 1834.

---

27. An Inquiry into the Nature of Sleep and Death, with a View to ascertain the more immediate Causes of Death, and the better Regulation of the Means of obviating them. (Re-published from the Philosophical Transactions.) By A. P. W. Philip, M.D. F.R.S. Octavo, pp. 254. 1834.

